# A Mathematical Framework for the Selection of an Optimal Set of Peptides for Epitope-Based Vaccines

**DOI:** 10.1371/journal.pcbi.1000246

**Published:** 2008-12-26

**Authors:** Nora C. Toussaint, Pierre Dönnes, Oliver Kohlbacher

**Affiliations:** Simulation of Biological Systems, Center for Bioinformatics, Eberhard Karls University Tübingen, Tübingen, Germany; Utrecht University, Netherlands

## Abstract

Epitope-based vaccines (EVs) have a wide range of applications: from therapeutic to prophylactic approaches, from infectious diseases to cancer. The development of an EV is based on the knowledge of target-specific antigens from which immunogenic peptides, so-called epitopes, are derived. Such epitopes form the key components of the EV. Due to regulatory, economic, and practical concerns the number of epitopes that can be included in an EV is limited. Furthermore, as the major histocompatibility complex (MHC) binding these epitopes is highly polymorphic, every patient possesses a set of MHC class I and class II molecules of differing specificities. A peptide combination effective for one person can thus be completely ineffective for another. This renders the optimal selection of these epitopes an important and interesting optimization problem. In this work we present a mathematical framework based on integer linear programming (ILP) that allows the formulation of various flavors of the vaccine design problem and the efficient identification of optimal sets of epitopes. Out of a user-defined set of predicted or experimentally determined epitopes, the framework selects the set with the maximum likelihood of eliciting a broad and potent immune response. Our ILP approach allows an elegant and flexible formulation of numerous variants of the EV design problem. In order to demonstrate this, we show how common immunological requirements for a good EV (e.g., coverage of epitopes from each antigen, coverage of all MHC alleles in a set, or avoidance of epitopes with high mutation rates) can be translated into constraints or modifications of the objective function within the ILP framework. An implementation of the algorithm outperforms a simple greedy strategy as well as a previously suggested evolutionary algorithm and has runtimes on the order of seconds for typical problem sizes.

## Author Summary

Over the last decade the design of tailor-made vaccines for prophylactic applications (e.g., prevention of infection) and therapeutic applications (e.g., cancer therapy) has attracted significant interest. Epitope-based vaccines are good candidates for such tailor-made approaches. They trigger an immune response by confronting the immune system with immunogenic peptides derived from, e.g., viral- or cancer-specific proteins. These peptides bind to major histocompatibility complex (MHC) molecules in a specific manner. The resulting complex is crucial for immune system activation. However, there are many allelic variants of MHC molecules, meaning that different patients typically bind different repertoires of peptides. Nevertheless, due to economical and regulatory issues one cannot simply add all immunogenic peptides to such a peptide mix. Hence, it is crucial to identify the optimal set of peptides for a vaccine, given constraints such as MHC allele frequencies in the target population, peptide mutation rates, and maximum number of selected peptides. In this work we formalize this problem, and variants thereof, in a mathematical framework. The resulting optimization problem can be solved efficiently and yields a provably optimal peptide combination. We can show that the method performs better than existing solutions. Furthermore, the framework is highly flexible and can easily handle additional criteria.

## Introduction

The development of vaccines and their subsequent large-scale prophylactic use was undoubtedly one of the most important developments in medicine. Vaccines make use of the adaptive part of the human immune system to protect from future infections (e.g., prophylactic vaccines used against viruses) as well as to fight chronic diseases and cancer.

Cellular adaptive immunity is, at its core, triggered by the recognition of immunogenic peptides bound to MHC class I (MHC I) and II molecules by T-cell receptors located on the surface of T cells. These peptides are derived from antigens, i.e., proteins that can cause an immune response, as a result of rather complex antigen processing pathways *in vivo*. Peptides capable of causing such an immune response are called epitopes and represent the smallest subunits that may be used therapeutically.

There are numerous options for constructing a vaccine once a set of potential antigens is known. The antigens or parts thereof can be used as intact proteins [1],[2], they can be administered as RNA or DNA coding for the antigen [3],[4], or the epitopes contained in the antigens may be used for vaccines. As discussed in detail in [5] the use of epitope-based vaccines (EVs) brings about manifold advantages, e.g., safety, ease of production, analytical control, and distribution. Skilled selection of epitopes can precisely direct the evoked immune response at conserved and highly immunogenic regions of several antigens. Due to these advantages and the applicability in personalized vaccination, EVs have recently been getting more and more attention. The recent review of EVs by Purcell *et al.* [5] gives a good overview of the state of the art as well as its achievements. We will thus only point out some recent studies.

EVs have proven successful in preclinical trials in mice [6], on which many of the preliminary studies have been conducted. A large number of clinical studies, both from academia and industry, have also been successful and have entered and/or completed clinical phase I and II trials [7]–[11]. Several commercial products have now entered clinical phase III trials. The indications for the vaccines in trial are mostly various cancers (e.g., leukemia, colorectal cancer, gastric cancer, lung cancer) and infectious diseases (predominantly HIV and hepatitis C virus).

The design of an EV entails one critical step, the selection of the epitopes. From the set of antigens, one can experimentally determine or computationally predict epitopes for a variety of MHC alleles. The crucial task is to select the set of epitopes which yields the best immune response in a given population while at the same time keeping the number of epitopes low. This step is of course critical to the success of the vaccine. The selection is usually made on a case-by-case basis considering key properties for each epitope: overall immunogenicity, mutation tolerance, population coverage, antigen coverage, and antigen processing.

The selection methods used by the pharmaceutical industry are manifold. In 2004, Singh-Jasuja *et al.* presented the Tübingen approach [12] to acquire an experimentally validated initial list of epitopes from tumor associated antigens. In this work, the incorporation of computational methods for prediction of MHC-peptide binding in the process is proposed. Since they help to reduce the number of experiments to be performed, such prediction methods have become standard tools in immunology. Commonly used methods are SYFPEITHI [13], HLA_BIND/Bimas [14], SVMHC [15], NetMHC [16]–[18], EpiMatrix [19], and the methods [20]–[23] provided by the Immune Epitope Database [24].

Given the set of candidate peptides, computational methods can be used to determine the relevant attributes of each candidate. However, the final choice of the set of epitopes to be used in the vaccine is typically performed manually. Several groups have addressed this problem computationally. In 2005, DeGroot *et al.* [25] published an approach to creating highly immunogenic and conserved epitopes to be used in EVs. The authors use EpiMatrix [19] to estimate the MHC class II binding affinity of highly conserved 9mers from HIV-1 proteins. Peptides with high binding affinities are then used to construct extended peptides containing multiple overlapping 9mers. *In vitro* evaluation of the immunogenicity of a selected set of these extended peptides yielded positive results.

Recently, Vider-Shalit *et al.* [26] proposed using a genetic algorithm to design an ordered sequence of epitopes to be used in an EV. Information on peptide conservation and similarity to self-peptides is used to pre-filter the set of candidates, while information on MHC allele frequencies is used to select alleles of interest. The scoring function used for the heuristic takes into account the number of covered MHC alleles, the number of covered antigens, the number of covered MHC/antigen combinations, and the probability of each epitope to be properly cleaved in the sequence.

Two related approaches were published by Fischer *et al.* [27] and Nickle *et al.* [28]. Both groups focus on designing vaccine antigens capable of protecting against diverse HIV-1 strains. In order to do so, they use computational methods to compress the variation found in naturally occurring antigens into a small number of composite antigens.

**Figure 1:**
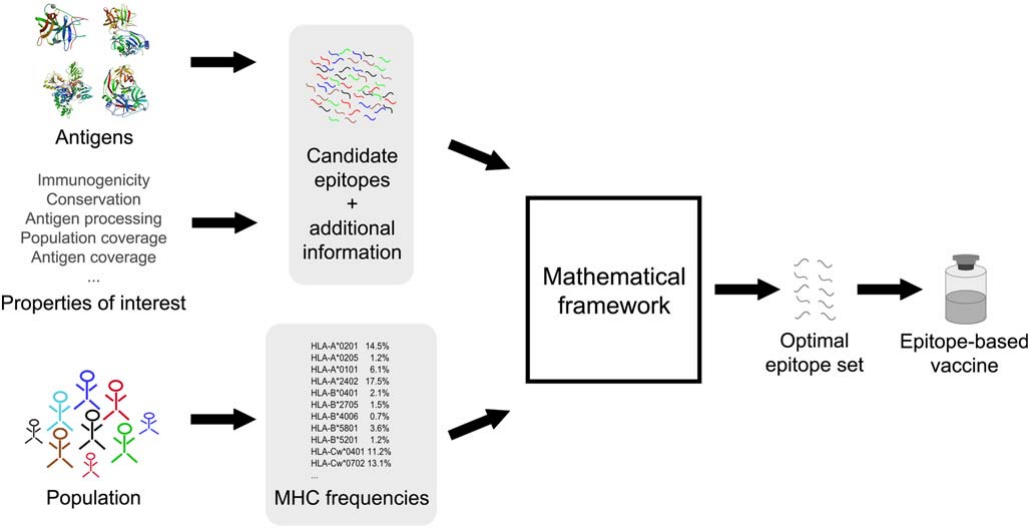
Basic idea behind this work. Starting from target antigens, a list of properties of interest, and a target population the information necessary to determine an optimal set of epitopes is derived (gray boxes). Given this information, a mathematical framework can conveniently be used to find the set of epitopes that is optimal with respect to the target population and the properties of interest.

Common to the computational approaches above (and of course manual selection) is the fact that the solutions are not necessarily optimal. None of the approaches can guarantee that there is not a better vaccine possible from the given set of epitopes. In this work we propose an integer linear programming approach to finding a provably optimal set of epitopes for an EV. Given a set of candidate peptides, a set of MHC alleles of interest, information on the peptides' respective immunogenicities along with other information to be incorporated into the selection process, our framework is capable of finding the set of epitopes yielding the highest possible overall immunogenicity (Figure 1). The resulting integer linear program can be solved very efficiently for all practical problem sizes (runtimes of a few seconds) and can thus be readily applied. With respect to various quality criteria (population coverage, antigen coverage, overall immunogenicity), the approach outperforms a simple greedy heuristic (‘pick the *k* best epitopes’) and also a genetic algorithm. The elegant mathematical formulation turns out to be flexible enough to also account for variants of the problem, e.g., the application to personalized vaccines. To our knowledge, this is the first epitope selection framework that finds the optimal solution.

## Materials and Methods

### Approach

In order to find an optimal set of epitopes, we first have to define what characterizes a *good* vaccine or, correspondingly, a *good* set of epitopes. This issue is highly controversial in the literature and only large-scale data from vaccination trials will provide sufficient data to validate the different approaches retrospectively. With this in mind, we do not suggest one optimal epitope selection strategy, but instead suggest a mathematical framework that allows working with various definitions of the term ‘good vaccine’. For a chosen definition, however, the algorithms will yield a combination of epitopes that is provably optimal.

In the following, we will introduce a ‘reasonable’ definition of a good vaccine. This will allow us to present the mathematical formulation and to illustrate how immunological requirements can be translated into mathematical constraints. For specific applications, the requirements and constraints may of course deviate from those given. For example, sequence variation in an antigen would be much more important for an HIV vaccine than for a cancer vaccine. The framework is flexible enough to allow for such different requirements, as we will illustrate towards the end of the work.

A good vaccine displays a high overall immunogenicity, which means it is capable of inducing potent immunity in a large fraction of the target population. This basic definition forms the basis of our mathematical formulation which aims at maximizing overall immunogenicity of the selected epitopes. We extend this definition by additionally requiring high mutation tolerance as well as a certain degree of allele and antigen coverage. Furthermore, the selected epitopes should display a high probability of passing through the antigen processing pathway. We thus obtain a brief list of basic requirements:

#### Mutation tolerance

Mutations within the targeted antigen regions can lead to an escape from immune response. High genetic variability as observed in, e.g., HIV, the hepatitis C virus, and influenza can thus affect protection by a vaccine. Selection of highly conserved non-overlapping epitopes reduces the chance of immune escape.

#### Allele coverage

Because the MHC is polygenic, every individual possesses a set of MHC loci. Due to the high polymorphicity of these loci, the pool of MHC molecules varies from individual to individual. The allelic form of an MHC molecule determines the spectrum of peptides the molecule can bind. Within a population alleles occur with different frequencies. Hence, requiring a certain number of alleles to be covered is equivalent to requiring a certain degree of population coverage. An MHC allele is said to be covered by a set of epitopes if at least one of the epitopes is capable of inducing an immune response when bound to the corresponding MHC molecule.

#### Antigen coverage

The expression frequencies of viral proteins differ. Selecting epitopes from a wide variety of antigens increases the chance of detecting a virus at any developmental stage.

#### Antigen processing

Before a peptide is presented by an MHC molecule on the cell surface it passes through an antigen processing pathway, which includes proteasomal cleavage and TAP transport. Knowledge of these steps' specificities allows exclusion of peptides which are unlikely to ever be presented to a T cell.

From all possible epitope combinations, the ones with a maximum overall immunogenicity will be called ‘optimal’ (there may be more than one optimal epitope combination). Hence, the search for an optimal epitope set for an EV can be interpreted as an optimization problem: out of a given set of epitopes, choose a subset which, out of all subsets meeting the above-named requirements, displays maximum overall immunogenicity. Since health agencies such as the FDA require proof of the effectiveness and safety of every individual component of a vaccine, the size of such a subset is usually kept rather small (up to a dozen peptides).

### Mathematical Abstraction

Given a set of epitopes and a set of MHC alleles we assume a linear relationship to exist between (a) the immune response induced by all epitopes with respect to all alleles and (b) the immune responses induced by every single one of the epitopes with respect to each of the alleles. Thus, the overall immunogenicity of a set of epitopes depends on the immunogenicity of its components with respect to the different MHC alleles. Furthermore, the contribution of an allele directly depends on its probability of occurring within the target population. (In this context *probability* is commonly referred to as *frequency*. We use *probability* since it is the mathematically correct term.) More common alleles are weighted more than uncommon ones. Thus, overall immunogenicity *I* can be derived mathematically as a weighted sum over immunogenicities of epitopes *E* with respect to the given MHC alleles *A*:
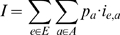
where *p_a_* corresponds to the probability of allele *a* in the target population and *i_e_*_,*a*_ to a measure of the immunogenicity of epitope *e* when bound to allele *a* (either experimentally determined or predicted).

### Integer Linear Programming

Our goal is to maximize overall immunogenicity while constraining the possible solutions to sets of peptides which satisfy the above mentioned requirements for a good vaccine. This problem can be conveniently formulated as an integer linear program (ILP). Linear programming deals with the optimization of linear objective functions subject to linear constraints [29]. An ILP is a linear program with integral unknowns. While linear programs without integral unknowns can be solved efficiently, ILPs are NP-complete. Nevertheless, there are tools available that find optimal solutions quite efficiently.

We restate the problem of choosing the optimal set of epitopes as an ILP. Solving the ILP will render an optimal solution according to our definition of an optimal epitope set. (Adapting the ILP to a different definition is straightforward.) For the sake of clarity, we start out with the very basic definition of an optimal epitope set. In the next step the resulting ILP will be extended to represent the more refined definition.

The set of candidate epitopes is *E*. Each epitope *e*∈*E* is associated with a binary decision variable x_e_, where *x_e_* = 1 if the respective epitope belongs to the optimal set and *x_e_* = 0 otherwise. The ILP corresponding to the basic definition of an optimal epitope set is shown in Table 1. This ILP maximizes overall immunogenicity: epitope immunogenicity with respect to a specific MHC allele is weighted by the allele's probability. The only constraint is the number of epitopes to select.

**Table 1:** ILP corresponding to the basic definition of an optimal epitope set.

**Definitions**	
*A*	Set of observed MHC alleles
*E*	Set of candidate epitopes
**Parameters**	
*i_e_*_,*a*_	Immunogenicity of epitope *e* with respect to allele *a*
*k*	Number of epitopes to select
*p_a_*	Probability of MHC allele *a* occurring in the target population
**Variables**	
x_e_ = 1	If epitope *e* belongs to the optimal set, otherwise x_e_ = 0
**Integer Linear Program**	
*Maximize*	Maximize …
∑*_e_*_∈*E*_x_e_∑*_a_*_∈*A*_ *p_a_ i_e_*_,*a*_	… Overall immunogenicity
*subject to*	
∑*_e_*_∈*E*_x_e_ = *k*	… And select exactly *k* peptides.

We will now extend this basic ILP to represent a more refined definition of a good epitope set. In order to implement the additional requirements we introduce another set of binary decision variables: each MHC allele *a* is associated with a variable y_a_. If allele *a* is covered, meaning that an epitope which is sufficiently immunogenic with respect to allele *a* belongs to the optimal set, y_a_ = 1, otherwise y_a_ = 0. The extended ILP is shown in Table 2. It accounts for mutation tolerance by selecting only non-overlapping conserved epitopes, and for allele and antigen coverage. Additional constraints prevent the selection of peptides which are unlikely to result from antigen processing.

**Table 2:** ILP corresponding to the extended definition of an optimal epitope set.

**Definitions**		
*A*	Set of observed MHC alleles	
*E_i_*	Set of epitopes from the *i*-th antigen	
*E*	Set of all candidate epitopes (*E* = *E*_1_ ∪…∪ *E_n_*)	
*I_a_*	Set of epitopes which, when bound to an MHC allele *a*, display an immunogenicity greater than or equal to a given threshold *τ^I^*	
*I*	Set of all sufficiently immunogenic epitopes (*I* = ∪*_a_*_∈*A*_ *I_a_*)	
*O*	Set of overlapping pairs of epitopes	
**Parameters**		
*c_e_*	Conservation of epitope *e*	
*i_e_*_,*a*_	Immunogenicity of epitope *e* with respect to allele *a*	
*k*	Number of epitopes to select	
*p_a_*	Probability of MHC allele *a* occurring in the target population	
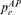	Probability that epitope *e* will be produced during antigen processing	
*τ^A^*	Minimum number of epitopes from each antigen to be included	
*τ^AP^*	Antigen processing threshold	
*τ^C^*	Conservation threshold	
*τ^MHC^*	Minimum number of MHC alleles to be covered	
**Variables**		
x_e_ = 1	If epitope *e* belongs to the optimal set, otherwise x_e_ = 0	
y_a_ = 1	If allele *a* is covered by the optimal set, otherwise y_a_ = 0	
**Integer Linear Program**		
	*Maximize*	Maximize …
	∑*_e_*_∈*E*_x_e_∑*_a_*_∈*A*_ *p_a_ i_e_*_,*a*_	… Overall immunogenicity.
	*subject to*	
	∑*_e_*_∈*E*_x_e_ = *k*	Selects exactly *k* epitopes.
∀*e*∈*E*:	(1−*c_e_*)x_e_≤(1−*τ^C^*)	Ensures certain degree of epitope conservation.
∀(*p*, *r*)∈*O*:	x_p_+x_r_≤1	Guarantees that selected epitopes do not overlap.
∀*a*∈*A*:	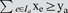	Guarantees that y_a_ = 1 only if allele *a* is covered.
	∑*_a_*_∈*A*_y_a_≥*τ^MHC^*	Enforces required allele coverage.
∀*i*∈{1,…,*n*}:	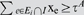	Enforces required antigen coverage.
∀*e*∈*E*:	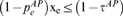	Selects only epitopes which have a chance of at least *τ^AP^* to result from antigen processing.

It might be desirable to obtain several optimal or nearly optimal epitope sets. As proposed in [30], this can be achieved by adding the constraints given in eq. (1), where *S_j_* represents the optimal set of epitopes found in iteration *j* and *s* represents the number of solutions to be obtained. The ILP has to be solved iteratively *s* times. After each iteration, the ILP for the next iteration *j*+1 is created by adding the corresponding constraint to the ILP of iteration *j*.
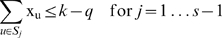
(1)


Every resulting epitope set differs from all other solutions in at least *q* peptides, 1≤*q*≤*k*.

### Nonlinear Requirements

In order to incorporate a requirement into the ILP framework it must be formulated as a linear constraint. There are, however, reasonable requirements which are non-linear. These cannot be incorporated directly. It is possible though to search a sufficiently large set of optimal and suboptimal solutions for the best set of epitopes that yields the required properties—provided that the requirements are feasible. Two examples of reasonable non-linear requirements will be discussed below.

#### Example 1: Population coverage

A major interest in vaccine design is population coverage: For what fraction of a target population will the resulting EV be effective? In theory this corresponds to the probability of an individual in the population carrying at least one MHC allele covered by the epitopes in the EV. Given a set of MHC alleles *A* as before and their distribution within a population, the population coverage of a particular set of epitopes can be computed. For this computation the polygenicity of the MHC has to be taken into account. It is *A* = *A*_1_ ∪…∪ *A_m_* with *A_i_* being the alleles at locus *i*. Let 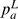 be the probability of an allele *a* occurring at the corresponding MHC locus. Then the probability of an individual in the population carrying an allele from the set *A_i_* at locus *i* corresponds to
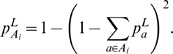
Let y_a_ be as described above. It follows that the probability of an individual carrying at least one MHC allele covered by the epitopes in *E*, and thus the population coverage of *E*, is
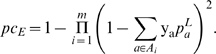



#### Example 2: Average number of epitopes per individual

Population coverage of an epitope set states what fraction of a population carries an MHC allele associated with one of the epitopes. It does not give any information on the number of active epitopes per individual. The number of epitopes within a set which are active for a specific individual depends on the individual's MHC genotype. Given the haploidic probabilities of MHC alleles within a population the probability of MHC genotypes can be calculated. Alleles not included in the set *A* are accounted for by adding a representative allele *X* to each locus. The frequency of the representative at locus *i* results from 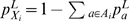.

Let *G* be the set of genotypes within the population of interest and *p_g_* the probability of genotype *g*. Furthermore, let *b_g_* be the number of epitopes in an epitope set *E* which are immunogenic with respect to an MHC allele in *g*. The average number of active epitopes per individual results from
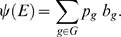



### Evaluation

Vider-Shalit *et al.* [26] applied their evolutionary-algorithm-based vaccine design method to hepatitis C virus (HCV). We used our framework on similar data and compared the results of both approaches.

#### Data

HCV protein sequences (amino acid frame 1) for ten different proteins (C, E1, E2, p7, NS2, NS3, NS4A, NS4B, NS5A, NS5B) and four different subtypes (1a, 1b, 2a, 3a) were retrieved from the Los Alamos hepatitis C sequence database [31]. For each protein of each subtype a multiple sequence alignment (MSA) was created using MUSCLE [32], resulting in 40 MSAs. From each MSA a consensus sequence was created. All 9mers from these consensus sequences were regarded as potential epitopes. *In silico* predicted MHC binding affinities using BIMAS matrices [14] are utilized as a measure of immunogenicity. MHC alleles, their probability of occurring in the human population, and binding affinity score thresholds were directly taken from Vider-Shalit *et al.* To allow a comparison of our results with those of Vider-Shalit *et al.*, we adopt their simplistic definition of peptide conservation (A peptide is considered to be at least *x*% conserved if all of its amino acids display a conservation of at least *x*%.) and disregard all insufficiently conserved (<90%) peptides. To score the probability of a peptide being a result of antigen processing, we used the proteasomal cleavage matrix from the supplementary material of [26]. As noted in several places, the influence of TAP transport is often rather limited [26],[33]. Consideration of TAP transport is thus omitted for this example.

It has to be noted that the accuracies of the prediction methods cause some limitations. MHC-peptide binding can be predicted with relatively high accuracy for many alleles, whereas proteasomal cleavage prediction leaves more room for improvement.

#### Incorporating the scoring function

The scoring function *S* of Vider-Shalit *et al.* takes into account the number of covered MHC alleles, the number of covered antigens, the number of covered MHC/antigen combinations, and a score for the probability of each epitope in the ordered sequence being properly cleaved:
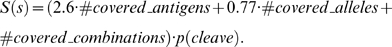
Here, *s* represents the ordered sequence of epitopes to be scored. In order to show the flexibility of our approach we incorporate aspects of this function in our ILP. Since the aim of our framework is to select a set of epitopes and not to create an epitope sequence, we omit the factor *p*(*cleave*).

Binary variables have to be introduced in order to count the number of covered antigens and the number of covered MHC/antigen combinations: z_i_ = 1 if an epitope from the *i*-th antigen belongs to the optimal set and z_i_ = 0 otherwise. w_a,i_ = 1 if an epitope from the *i*-th antigen, which is sufficiently immunogenic with respect to MHC allele *a*, belongs to the optimal set and w_a,i_ = 0 otherwise. Since immunogenicity scores tend to be higher than the weighted sums of the coverage scores and would therefore outweigh them, we scale the immunogenicity by a (purely empirical) factor of 0.1. The resulting ILP still aims at high overall immunogenicity while at the same time extending the coverage of antigens, MHC alleles, and MHC/antigen combinations. The ILP is shown in Table 3.

**Table 3:** ILP corresponding to the combined optimization problem.

**Definitions**		
*A*	Set of observed MHC alleles	
*E_i_*	Set of epitopes from the *i*-th antigen	
*E*	Set of all candidate epitopes (*E* = *E*_1_ ∪…∪ *E_n_*)	
*I_a_*	Set of epitopes which, when bound to an MHC allele *a*, display an immunogenicity greater than or equal to a given threshold *τ^I^*	
*I*	Set of all sufficiently immunogenic epitopes (*I* = ∪*_a_*_∈*A*_ *I_a_*)	
**Parameters**		
*i_e_*_,*a*_	Immunogenicity of epitope *e* with respect to allele *a*	
*p_a_*	Probability of MHC allele *a* occurring in the target population	
**Variables**		
w_a,i_ = 1	If allele *a* is covered by an epitope from the *i*-th antigen, otherwise w_a,i_ = 0	
x_e_ = 1	If epitope *e* belongs to the optimal set, otherwise x_e_ = 0	
y_a_ = 1	If allele *a* is covered by the optimal set, otherwise y_a_ = 0	
z_i_ = 1	If an epitope from the *i*-th antigen belongs to the optimal set, otherwise z_i_ = 0	
**Integer Linear Program**		
	*Maximize*	Maximize …
	0.1·∑*_e_*_∈*E*_x_e_∑*_a_*_∈*A*_ *p_a_ i_e_*_,*a*_ +	… Overall immunogenicity and …
	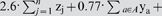	… Extend coverage of antigens, MHC alleles, …
	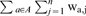	… And MHC/antigen combinations.
	*Subject to*	
	All constraints from the extended ILP (Table 2)	
∀*i*∈{1,…,*n*}	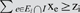	Ensures that z_i_ = 1 only if the *i*-th antigen is covered.
∀*i*∈{1,…,*n*} *a*∈*A*	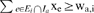	Ensures that w_a,i_ = 1 only if allele *a* is covered by an epitope from the *i*-th antigen.

### Implementation

We used ILOG CPLEX 9.1 [34] with its C++ interface ILOG Concert Technology 2.1 to formulate and solve the ILP. It is, however, possible to solve the ILPs with most other ILP solvers, e.g., MOSEK [35] or freely available packages like SCIP [36],[37].

A formulation of the extended ILP (Table 2) as ILOG CPLEX input, the required data for the comparison with Vider-Shalit *et al.* [26] as well as the corresponding ILOG CPLEX output can be found in the supplementary material (Texts S1, S2, S3).

## Results

### Immunogenicity

In order to show the effectiveness of the above-mentioned approach, we compare our strategy with other published approaches and determine the theoretical gain in immunogenicity or the number of epitopes required to achieve a similar immunogenicity. While an experimental validation of this approach would be valuable, it is beyond the scope of this paper, which focuses on the theoretical foundations of the epitope selection. We compare our optimal strategy (best overall immunogenicity, BOI) with two simple approaches:

- randomly select *k* peptides out of a pool of good epitopes (random set of epitopes, RSE) and
- a simple greedy approach: pick the *k* best epitopes from the set (best epitope-wise immunogenicity, BEI).

**Figure 2:**
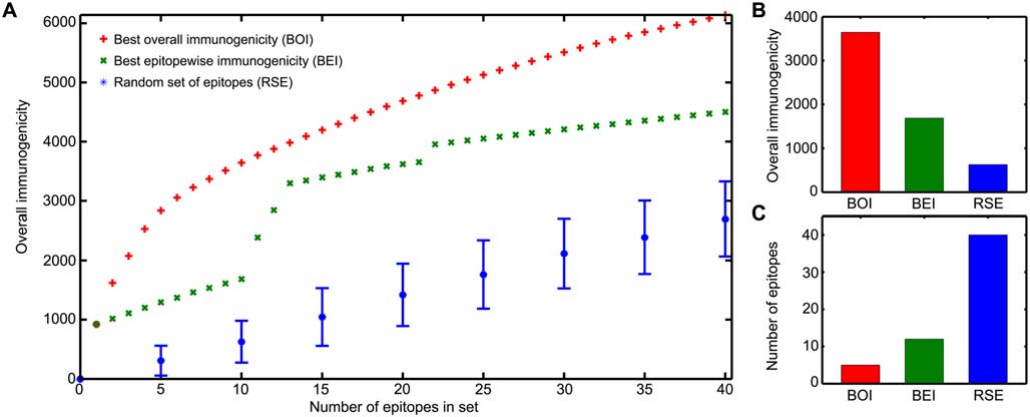
Comparison of different epitope selection strategies with respect to overall immunogenicity. (A) Overall immunogenicity of different-sized epitope sets. (B) Overall immunogenicity of a set of 10 epitopes. (C) Number of epitopes required to achieve an overall immunogenicity of at least 2,699.

These three epitope selection strategies were used to select different-sized sets of epitopes from a set of 4461 conserved (≥90%) HCV 9mers. For BOI the basic ILP (Table 1) was used to maximize overall immunogenicity. BEI selects the epitopes with the highest sum of immunogenicities irrespective of the probabilities of the corresponding MHC alleles. The overall immunogenicity of each epitope set was determined and is displayed in Figure 2. For RSE, mean and standard deviation of 100 random selections of different-sized epitope sets from the 100 most immunogenic peptides are shown. The BEI curve shows sudden increases in overall immunogenicity from 0 to 1, 10 to 13, and from 20 to 21 epitopes. This is caused by the selection of epitopes which are highly immunogenic with respect to HLA-A*0201, which is the most common (*p^L^* = 0.145) among the considered alleles. All other selected epitopes are highly immunogenic with respect to less common alleles like HLA-B*2705 (*p^L^* = 0.015) or HLA-B*5102 (*p^L^* = 0.003). Thus the former contribute more extensively to the overall immunogenicity than the latter.

The average overall immunogenicity of the randomly chosen epitope sets is rather low: scores range from about 308 for five epitopes, to 1,763 for 25 epitopes, to 2,699 for 40 epitopes. The other two approaches start from a minimum overall immunogenicity of more than 900 and reach immunogenicities of 4,502 (BEI) and 6,142 (BOI), respectively. To achieve an immunogenicity of at least 2,699, BOI requires five and BEI 12 epitopes (Figure 2).

For sets with more than one epitope, the scores yielded by the BOI strategy are between about 20% (13 epitopes) and 120% (6 epitopes) higher than those of the BEI strategy.

### Comparison with Vider-Shalit *et al.* on HCV

In order to compare our approach to the work of Vider-Shalit *et al.* [26] we applied the ILP given in Table 2 to the HCV data and 27 of the 29 alleles from [26]. The alleles HLA-B^*^0702 and HLA-B^*^3501 were omitted, since none of the candidate peptides binds to them. Probably due to an error in sequence processing (personal communication with Yoram Louzoun), a peptide (AALENLVTL) which does not belong to any of the proteins under consideration was included in the 25 epitopes selected by Vider-Shalit *et al.* We exclude this peptide and base our comparison on sets of 24 epitopes.

For the 24 epitopes to be selected, we require a minimum conservation of 90%, an allele coverage of 27, and an antigen coverage of at least one epitope per antigen. Furthermore, only epitopes with antigen processing scores within the top 30% of all sufficiently conserved candidate peptides were allowed to be selected. The following 24 epitopes (hereafter *E_ILP_*) were selected:

**Table:** 

SFSIFLLAL^*^	GHRMAWDMM^+^	VYEADDVIL
YLYDHLAPM	GLRDLAVAV^+^	GPTPLLYRL^+^
QYLAGLSTL^+^	NFVSGIQYL	VLSDFKTWL^*^
GLYLFNWAV	ALYDVVSTL^*^	RRCRASGVL^+^
CFTPSPVVV^+^	FLLLADARV^*^	GPADGMVSK^+^
TWVLVGGVL^+^	IELGGKPAL^+^	LAGGVLAAV
ARPDYNPPL^+^	KLLPRLPGV	RHTPVNSWL^+^
WPLLLLLLA	VTYSLTGLW	YFVIFFVAA

Four epitopes (marked with *) are known HCV epitopes and can be found in the Immune Epitope Database (IEDB, release 2008_4_1_3_28) [24]. Another 11 epitopes (marked with +) are contained in known longer epitopes. The overall immunogenicity of the selected set is 2,549. It includes binders for all 27 alleles with all 40 antigens being represented and covers 22.7% of all MHC/antigen combinations. The average number of epitopes per individual of the population is 13.3. The corresponding values of the epitope set selected by Vider-Shalit *et al.* (hereafter *E_VS_*) are listed in Table 4.

**Table 4:** Overview over properties of HCV epitope sets selected using different strategies.

	*E_ILP_*	*E_VS_*	*E_Comb_*
Overall immunogenicity	**2,549**	125	2,177
Allele coverage	**100%**	96.3%	**100%**
Antigen coverage	**100%**	87.5%	**100%**
MHC/antigen coverage	22.7%	19.2%	**30.5%**
Population coverage	**96.0%**	95.6%	**96.0%**
Avg. number of epitopes per individual	13.3	11.4	**17.3**
Number of epitopes in IEDB	**4**	1	1

Number of epitopes per set: 24. *E_ILP_*: set selected by our ILP, *E_VS_*: set selected by Vider-Shalit *et al.* without peptide AALENLVTL, *E_Comb_*: set selected by our ILP extended by aspects of the scoring function of Vider-Shalit *et al.*

**Figure 3:**
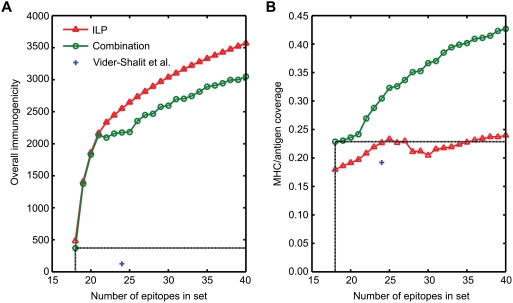
Comparison of properties of HCV epitope sets selected using different strategies. (A) Overall immunogenicity. (B) Coverage of MHC/antigen pairs.

To improve the MHC/antigen coverage while still aiming at high overall immunogenicity we included the central part of the scoring function of Vider-Shalit *et al.* in the objective function of our ILP. The optimal epitope set with respect to the modified objective function (hereafter *E_Comb_*) is only 15% less immunogenic than the original epitope set *E_ILP_* and more than 17 times more immunogenic than *E_VS_*. As for MHC/antigen coverage, it outperforms both (Table 4). *E_Comb_* includes one epitope which is already known and 14 epitopes which are contained in longer epitopes listed in the IEDB. Figure 3 shows that when using the combined objective function, 18 epitopes suffice to cover all alleles and antigens and furthermore to outperform *E_VS_* in terms of immunogenicity (371) and MHC/antigen coverage (22.8%).

## Discussion

The selection of an epitope set with very high overall immunogenicity is crucial for the efficacy of an EV. Depending on the number of candidate epitopes to choose from, the number of alleles to be considered, as well as on the additional requirements, this problem can become very complex. In this work we propose a mathematical framework that can be used to solve this problem quickly for practical problem sizes. For several characteristic examples, we show that immunological requirements can be conveniently formulated as an ILP. The solution of this ILP yields an optimal set of epitopes: the set of epitopes that displays the highest overall immunogenicity of all sets which meet the pre-defined requirements. To our knowledge, this is the only approach that yields provably optimal solutions to the vaccine design problem for EVs. In contrast to previous heuristics, the optimal solution yields either significantly better overall immunogenicity for the same number of epitopes or a smaller number of required epitopes to reach the same level of immunogenicity. The flexibility of the framework allows selecting other objective functions, too, for example, maximizing antigen or allele coverage.

The optimal selection of epitopes yields—in theory—significantly higher overall immunogenicities than other strategies (e.g., selection of the best epitopes or evolutionary algorithms). However, one should keep in mind that the selection of the epitopes is still a difficult and controversial issue since the underlying processes are not yet fully understood. In particular, the interplay of different epitopes poses a difficult problem. Competition between epitopes will probably result in reduced immunogenicity of peptide cocktails, an effect that has been observed in various studies.

On the one hand, this represents a problem, because the assumption of independence between epitopes is one of the key assumptions made in this work (and in all related approaches). Lacking an accurate model of these competition effects, however, it seems like the best assumption one can make. On the other hand, the effects of competition are a compelling reason to employ this type of selection approach. Competition effects will be less severe for fewer peptides, therefore a selection procedure that yields the same overall immunogenicity with fewer peptides can in fact mitigate this problem. Assuming that competition primarily arises between epitopes binding to the same MHC allele, one can also introduce additional constraints to reduce competition (e.g., find the best combination that contains at most two epitopes per allele). In the long run, a thorough quantitative analysis of larger vaccination studies might shed some light on these effects and their importance.

Also, the notion of immunogenicity alone, or the ability to evoke an immune response in a certain fraction of patients, is not necessarily a true measure of quality for a vaccine. In their recent review on the quality of the T-cell response [38], Seder *et al.* argue that protective T-cell responses are too complex to be sufficiently described by a measure of magnitude alone. An adequate metric would thus not only account for the magnitude but also for the multifunctional quality of the response. The flexibility of our framework allows for the incorporation of a different quality measure for immunogenicity and a careful comparison of the peptide cocktails suggested by different objective functions would be very interesting.

In their review of EVs, Purcell *et al.* [5] point out that, to date, there are no human EVs on the market. This is mainly attributed to the difficulties associated with peptide stability and delivery. Various delivery strategies [39] are being explored in clinical studies. In an extension of this work, one might therefore also include considerations related to the peptide delivery. For *beads-on-a-string* type vaccines, the selected epitopes are combined into one larger polypeptide. As the specificities of the antigen processing pathway have to be taken into account when constructing the polypeptide, the order of the epitopes as well as possible spacer sequences need to be optimized (e.g. through incorporation of a proteasomal cleavage matrix).

## Supporting Information

Text S1 ILOG CPLEX input. AMPL formulation of the extended ILP given in Table 2 adapted for the comparison with Vider-Shalit et al.
(3.00 KB TXT)

Text S2 HCV data (in AMPL format) used for the comparison with Vider-Shalit et al. Only highly conserved peptides (> = 90%) were considered when generating this file.
(1.70 MB TXT)

Text S3 ILOG CPLEX output.
(2.00 KB TXT)
